# Fabrication of disposable microextraction analytical tool for *in vitro* detection of *Staphylococcus* bacterial pathogen using volatile metabolites emission†

**DOI:** 10.1039/d4ra09099c

**Published:** 2025-04-10

**Authors:** Keerthana Selvamuthukumar, Debsmita Mandal, Harshika Poojary, Gouri Illanad, Bharath Prasad A. S., Sophia Koo, Chiranjit Ghosh

**Affiliations:** a Department of Biotechnology, Manipal Institute of Technology (MIT), Manipal Academy of Higher Education, Manipal (MAHE) Karnataka 576104 India chiranjit.ghosh@manipal.edu; b Department of Biotechnology, KLE Technological University Hubballi Karnataka 580021 India; c Department of Public Health Genomics, Manipal School of Life Sciences (MSLS), Manipal Academy of Higher Education, Manipal (MAHE) Karnataka 576104 India; d Division of Infectious Diseases, Brigham and Women's Hospital 181 Longwood Avenue, MCP642 Boston MA 02115 USA; e Harvard Medical School, Harvard University 25 Shattuck Street Boston 02115 MA USA; f Dana-Farber Cancer Institute 450 Brookline Avenue Boston MA 02215 USA

## Abstract

A disposable paper-based thin film solid-phase microextraction (TF-SPME) patch was developed for the detection of *Staphylococcus aureus* bacterial pathogen. The study was based on the extraction of volatile organic compounds from the bacterial culture medium by a nanoparticle blended polymer-coated microextraction patch and then analyzed by gas chromatography-mass spectrometry to identify the volatile metabolic signature associated with the bacterial pathogen during the growth phase of the bacterial species in the culture medium. The TF-SPME patches were fabricated using a divinylbenzene/multiwall carbon nanotube/polydimethylsiloxane coating mixture employing a film applicator for uniform coating on a regular cellular paper substrate. The coated sheet was dried and trimmed into multiple small-dimension sampling patches before exposure to the *Staphylococcus* bacterial solution. To check the eco-friendliness of the proposed technique in terms of green analytical chemistry, the ‘Blue Applicability Grade Index’ (BAGI) was determined to be around 62.5, suggesting the feasibility of considering the proposed analytical method as a green sample preparation approach for clinical application. Therefore, this technique utilizing the TF-SPME patches may be utilized as an alternative and rapid method for the identification of *Staphylococcus* bacterial pathogens as an alternative to the traditional prolonged culture-based study. Furthermore, the microextraction patch is disposable and easy to fabricate, suggesting the feasibility of utilizing it as a pathological sampling kit for the characterization of *Staphylococcus* bacterial pathogen.

## Abstract 2

Development of paper-based thin film microextraction for diagnosis of bacterial pneumonia.

## Graphical abstract

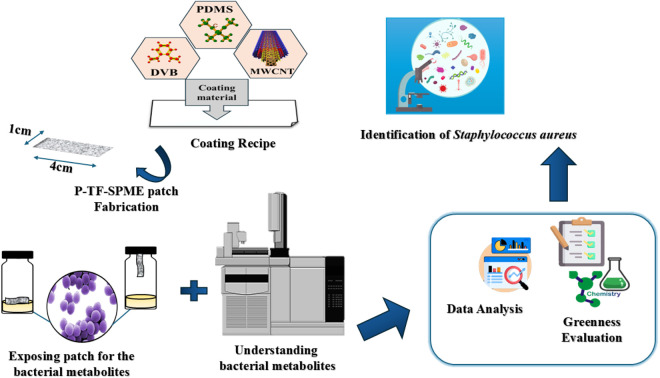

## 1. Introduction

Respiratory infection is a significant threat to patients due to the adaptability of the bacterial species in the hospital environment and the simultaneous high risk of antimicrobial resistance.^1^*Staphylococcus aureus*, a Gram-positive pathogen,^2^ is accountable for the wide range of bacterial infections in both community^3^ and hospital settings.^4^ The antibiotic-resistant *Staphylococcus aureus* (MRSA) infection poses a significant public health threat with an alarming mortality rate, especially among immunocompromised and critically ill patients.^5^ It is also responsible for ventilator-associated pneumonia (VAP), a medical condition in which a hospital patient develops pneumonia 48 hours after starting mechanical ventilation. *Staphylococcus aureus* has been recognized as a causative pathogen, accounting for 29.6% of VAP cases.^6^

The traditional method of *Staphylococcus* pathogen identification consists of culture of bacterial species in pathology, and it takes a few days to obtain information about the pathogen. Bacterial protein can be identified by the polymerase chain reaction (PCR). However, it is rarely available in remote healthcare settings, suggesting the current dependency on culture-based investigation for the identification of *Staphylococcus* pathogen in regular pathology in undeveloped and developing countries. The information regarding the bacterial species is important to clinicians for the effective treatment of patients with *Staphylococcus*-associated antimicrobial resistance.^7^ Therefore, there is a pressing need to develop a fast and potential technique for the characterization of *Staphylococcus* bacterial species from biological samples.

In recent years, solid-phase microextraction (SPME) has gained immense popularity as a green analytical sample preparation technique for preconcentrating volatile metabolites.^8,9^ SPME is a potential sample preparation approach for capturing volatile metabolites. It works on the principle of mass transfer of analytes during sampling due to the partition equilibrium between the sample matrix and the sorbent coating materials on the fibre substrate. However, the limitation of the traditional SPME fiber lies in its fragile nature and low extraction efficiency. To overcome the issues, researchers developed an advanced and alternative geometry of SPME through the conversion of the fiber to microextraction patches, known as “thin film solid-phase microextraction” (TF-SPME).^10^ The patches have high enrichment efficiency of analytes due to the large surface area-to-volume ratio in the sampling tool.^11,12^ The TF-SPME patch can extract metabolites by integrating the tool into the sample matrix through the direct immersion and headspace modes of extraction from the sample matrix.^13^ Researchers utilized the hydrophilic–lipophilic balanced materials-coated TF-SPME patches to examine the bronchoalveolar lavage (BAL) samples from the patients infected with *Staphylococcus aureus*. The study reported the presence of ethyl-2-methylbutyrate.^14^ Further study utilized TF-SPME with a two-dimensional air-liquid interface culture of *Staphylococcus aureus* and reported the phenylethyl alcohol from the infected cell of the pathogen.^15^ However, the commercial TF-SPME patches are expensive, and therefore, it is hardly possible to utilize those in a regular clinical setting. Therefore, there is a research gap in utilizing the TF-SPME to shorten the pathology-based sample preparation time for the characterization of bacterial pathogens.

To address this issue, this investigation demonstrated the development of a paper-based TF-SPME (p-TF-SPME) analytical sampling tool for the determination of *Staphylococcus aureus* bacterial species. To capture the volatile emission at trace levels from the culture media, the microextraction patches were coated with multi-walled carbon nanotubes (MWCNT), divinyl benzene (DVB) and polydimethylsiloxane (PDMS) for the extraction of semi-volatile and volatile compounds emitted by the pathogen.^16^ The developed p-TF-SPME patches were coupled to bacterial culture vials of *Staphylococcus* species for direct and headspace extraction of volatile metabolites from the sample matrix. After the extraction, the patches were desorbed with a small amount of organic solvent for mass transfer of the compounds from the patches to solvent and finally analyzed by the gas chromatography-mass spectrometry (GC-MS) to identify the *Staphylococcus*-emitted metabolites from the culture matrix. The proposed technique showed a considerable Blue Applicability Grade Index (BAGI) score, suggesting the contribution of this technique to the sustainable green chemistry approach. In future, the proposed p-TF-SPME could be utilized to design a pathology-based sampling kit for the characterization of the bacterial species as an alternative to the culture-based studies. This study may facilitate the rapid identification of bacterial pathogens responsible for respiratory infection caused by *Staphylococcus*.

## 2. Materials and methods

### 2.1. Chemicals and reagents

The chromatography-grade acetonitrile and hexane were procured from Sigma-Aldrich (US), whereas the ethanol was purchased from Loba Chemie Pvt Ltd (India). The monomer divinylbenzene (DVB) from Merck, 2,2-azobisisobutyronitrile (AIBN) initiator (Loba Chemie Pvt Ltd, India), polydimethylsiloxane (PDMS) and multi-walled carbon nanotube (MW-CNT) were purchased from Sigma-Aldrich, US. were utilized for the synthesis of the sorbent coating materials. Regular A4 size cellular papers (Copy Gold A4 sheet, GSM – 75, size 21 × 29.7 cm) were used as a substrate for fabricating the p-TF-SPME tool. The extractions were performed using 40 mL glass vials, which were purchased from Supelco (Product No.: 27180). The octane (CAS NO.: 114.23) was procured from Spectrochem Pvt Ltd (India) to validate TF-SPME patches while ethyl acetate was procured from Supelco. Luria–Bertani broth was purchased from BD Difco™. For the McReynolds standard solution was prepared using n-amyl alcohol and 2-pentanone (Loba Chemie Pvt Ltd, India), pyridine and benzene (Molychem), *n*-octane (Spectrochem Pvt Ltd, India) and 1-nitropropane (Sigma-Aldrich).

### 2.2. Instrumentation

During the synthesis of DVB, a magnetic stirrer (LMMS-5LC, Labman) and centrifuge Eppendorf 5804R were used. The uniform coating of the sorbent material was performed by Elcometer 4310 – an automatic film applicator (70 micrometre thickness). The extracted metabolites were analyzed using GC-MS-QP2020 NX SHIMADZU equipment. Field emission scanning electron microscopy (CARL ZEISS (USA), Model: SIGMA WITH GEMINI COLUMN) was used to study the morphological structure of the synthesized DVB. Furthermore, energy-dispersive X-ray analysis (EDAX) was utilized with the Bruker Nano XFlash detector for elemental analysis. X-ray diffraction (XRD) analysis was performed by Rigaku Miniflex 600 (5th gen) equipment with a scanning angle (2*θ*) of 1–80°,voltage of 40 kV and current of 15 mA.The thermal stability test of the patch was performed by the thermogravimetric analyzer, Simultaneous Thermal Analyzer (STA), STA7200 (Hitachi, Japan). The mechanical stability was checked by the Shimadzu-made Universal Testing Machine (UTM).

### 2.3. Ethical statements

Ethical clearance for this study was obtained from the Institutional Biosafety Committee (IBSC) of the Manipal Academy of Higher Education, Manipal.

### 2.4. Fabrication of paper-based thin film solid-phase microextraction patches

**Fig. 1:**
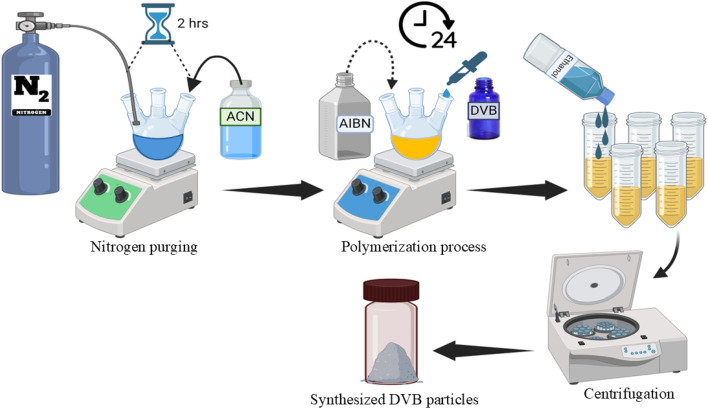
The synthesis of DVB polymer particles by precipitation polymerization process.

The development of p-TF-SPME patches involved a three-step process,^17^ which includes synthesizing DVB particles, coating the sorbent mixture onto A4 paper, and trimming the coated paper into desirable shapes or patches. The first step involved the synthesis of DVB particles using a precipitation polymerization technique, as represented in the Fig. 1. To synthesize the DVB polymer particles, the study was performed in a deoxygenated environment where 200 mL of acetonitrile (ACN) solution was added in a three-necked round-bottom flask equipped with a nitrogen purge system. To perform the polymerization process, DVB monomer (5 mL) was mixed with 2,2-azobisisobutyronitrile (AIBN) initiator to the ACN solvent in the flask. The mixture was stirred and heated overnight at 70 °C to initiate the polymerization reaction. The resultant solution was then centrifuged after the reaction to isolate and separate the DVB polymer particles. These particles were subsequently rinsed multiple times with ethanol and dried in a vacuum oven to make it powder form.

**Fig. 2:**
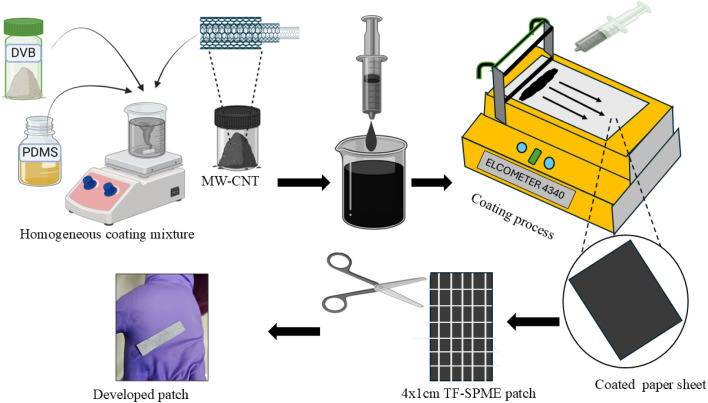
Fabrication of paper-based thin film solid-phase microextraction patches by utilizing a thin film applicator.

Next, the synthesized DVB particles (1 g) were used to prepare a composite coating material to fabricate the patches. The well-dispersed DVB particles were added to the mixture along with PDMS (3.6 g) as a binder and 1.3% MW-CNT (0.06 g). The PDMS was selected for its flexibility, chemical stability and compatibility with the DVB particles, while MW-CNT was included to enhance the mechanical strength and high surface area for the hydrophobic extraction. To ensure a homogenous mixture of the compounds, we vortexed the solution matrix for a long time. Later, to obtain a uniform coating, the A4 paper substrate was coated with a film applicator (Elcometer 4340). In this study, the coating thickness was kept at 70 μm. To remove the contaminants, the coated patches were dried in a nitrogen environment. The fabricated paper sheets were precisely trimmed into multiple TF-SPME patches with rectangular shapes of 4 cm × 1 cm dimension. The patches were then stored in a vacuum oven in a nitrogen environment for further investigation. Fig. 2 and S1† illustrate the coating process of the sorbent materials onto the paper.

### 2.5. Validation of p-TF-SPME patches

To compare the extraction efficiency and validate our patches, we extracted the analytes from the McReynolds standard (benzene, pyridine, octane, 1-pentanol, 2-pentanone and 1-nitropropane) by commercially available DVB/PDMS TF-SPME (manufactured by Markes International USA with batch no 640689) and the paper-based patches. To perform the experiments, the McReynolds standard was prepared at 1000 ng mL^−1^ concentration and directly exposed the individual patches into the solution matrix of 40 mL vials. The extraction was performed at a controlled temperature of 25 °C with a shaking speed of 120 rpm to ensure the efficient adsorption of the analytes onto the sorbent surface. Later, the desorption of analytes was carried out using 2 mL of acetonitrile at 40 °C with a shaking speed of 250 rpm in a shaking incubator. To validate the p-TF-SPME for headspace extraction, the McReynolds standard was used, whereas the efficiency of the patches for direct immersion was checked with an octane standard solution.

### 2.6. Microbiological works

In the experiment, the bacterial species *Staphylococcus aureus* (ATCC 29213) was used for the *in vitro* analysis of volatile metabolites. The glycerol stocks of the bacteria were thawed and inoculated into fresh Luria–Bertani broth (LB) before the preparation of samples for the analysis. After the overnight incubation at 37 °C, the bacteria was grown into the nutrient agar plates. A single isolated colony from the cultured plate was selected and then inoculated in 10 mL of LB broth in a separate vial. Later, the 2 mL culture media was aliquoted into five separate 40 mL vials. Before the inoculation, the sterilization was achieved by autoclaving the media at 121 °C for 15 min. The growth phase of *Staphylococcus* culture media was determined at OD 600 nm.

### 2.7. Preconcentration of volatile emission from *Staphylococcus aureus* culture media

**Fig. 3:**
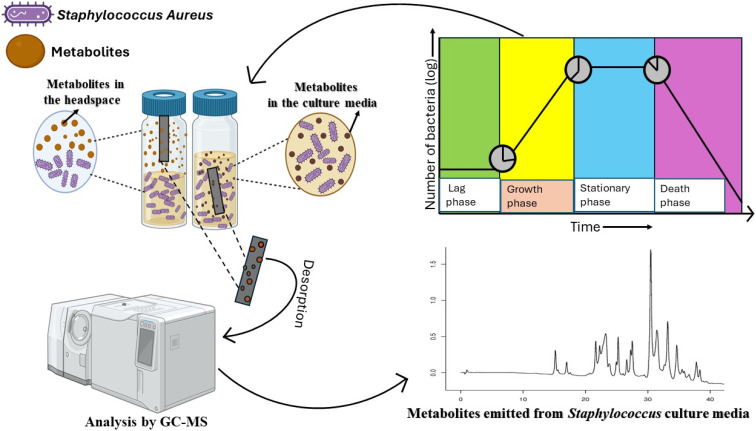
Extraction of the volatile organic compounds from the culture media of *Staphylococcus aureus* during the growth phase and analysis by the GC-MS.

The preconcentrated VOCs were extracted by the direct immersion (DI) and headspace (HS) sampling of the inoculated and control broths by the p-TF-SPME patches. The DVB/PDMS/CNT-coated patches were either directly immersed or exposed into the headspace of the vial for 1.5 h from the culture media. The exposure time was calculated based on the change in the optical density value of culture media from 0.1 to 0.5, corresponding to the growth phase of the *Staphylococcus* bacterial species.^18^ The patches were carefully removed and then desorbed with 1 mL of acetonitrile for 30 minutes. Fig. 3 and S2† demonstrate the experimental procedure for capturing the *Staphylococcus*-emitted metabolites from the culture media.

### 2.8. Analysis of metabolites by GC-MS

In the experiment, VOCs were captured by the p-TF-SPME patches and were analyzed using a single quadrupole GC-MS system. The column used for the study was the SH-I-5Sil MS with a dimension of 0.25 mm internal diameter and a length of 30 meters. Helium was utilized as a carrier gas with a consistent column flow rate of 1.20 mL min^−1^. The GC oven temperature was programmed as follows: initial temperature at 50 °C, hold for 2 min and then ramped with a rate of 8 °C up to 280 °C.

### 2.9. Green matrices

The greenness of the technique was assessed using the blue applicability grade index, software available at https://bagi-index.anvil.app/, with parameters listed in Table S1.†

### 2.10. Statistical analysis

The compounds were identified using the NIST library. Origin software was employed for statistical analysis of the data and the plots presented in the manuscript. The volatile compounds were matched with the literature and databases, including Biocyc, mVOC 3.0, MiMedb, to understand the relevance of the metabolites with the pathogen. The data was presented as mean ± standard error. Five replicate studies were performed to validate the patches. The reported volatile compounds from the cultured media were compared with the control study where the paper-based patches were exposed to the broth matrix without any bacterial solution.

## 3. Result and discussion

**Fig. 4:**
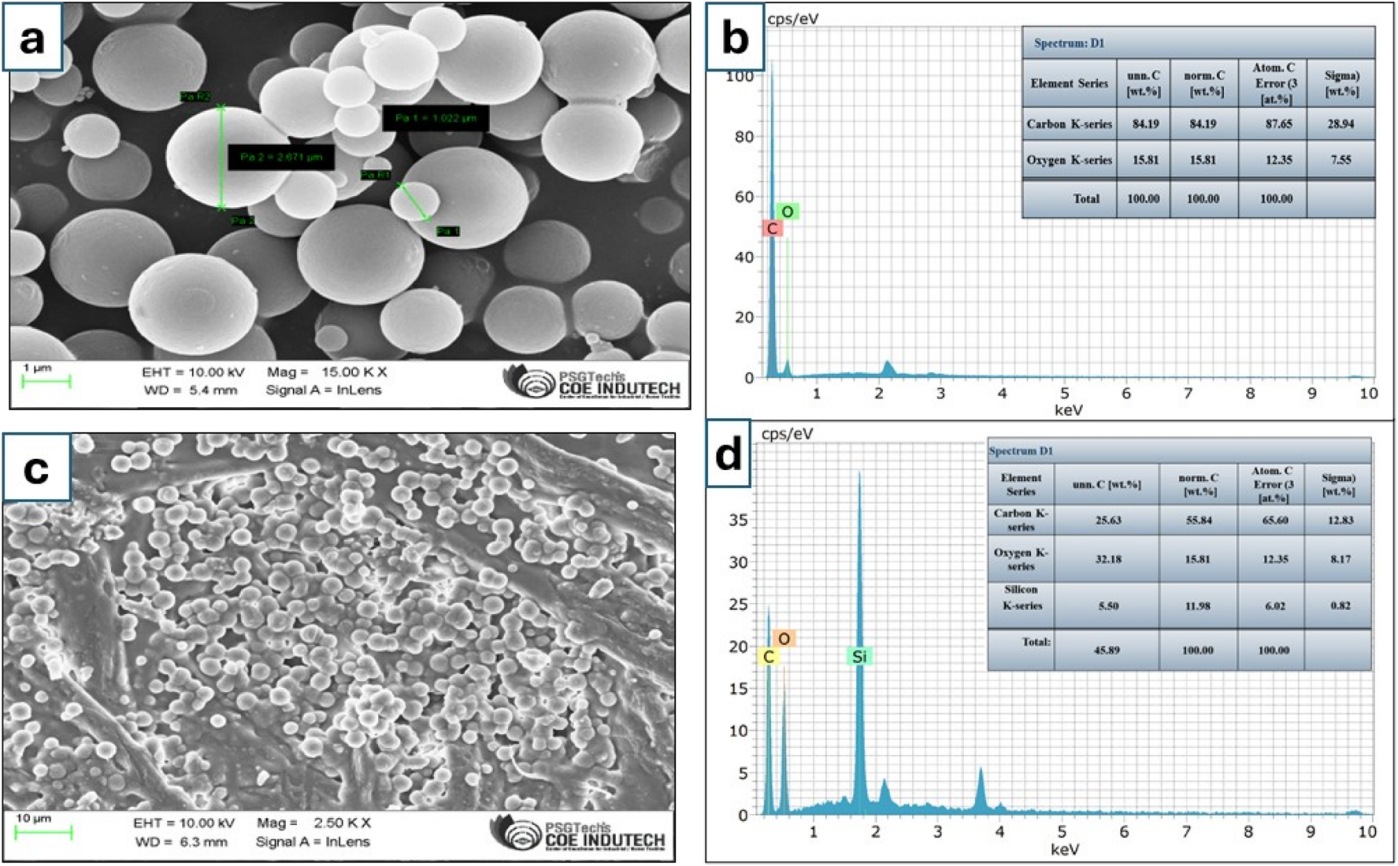
The morphological characterization of synthesized polymer DVB particles using FE-SEM (a) synthesized DVB polymer particle size ranged from 1–5 μm, (b) the EDX analysis of the synthesised polymer DVB (c) morphology of DVB/PDMS/CNT coated patches, and (d) the EDX analysis of the DVB/PDMS/CNT coated patches.

In this study, p-TF-SPME patches were developed for the characterization of *Staphylococcus* bacterial species through volatile emissions from the culture matrix. The FESEM data revealed that the DVB particles synthesized by our precipitation polymerization process were approximately 1–5 μm in diameter (Fig. 4a). The elemental data through EDX analysis confirmed that the synthesized DVB polymer particles were mainly composed of carbon and oxygen atoms with an elemental composition of 87.65% and 12.35%, respectively (Fig. 4b), suggesting that most of the particles were composed of carbon and a minor percentage of oxygen due to surface oxidation or impurities. The uniformity of the coating materials on the fabricated paper patches has been demonstrated in Fig. 4c. DVB contributes to the formation of a porous network, which can facilitate the adsorption of analytes by the particles, whereas the PDMS enhances the mechanical durability of the patch, with simultaneous diffusion of the analytes into the patch. The nanoparticle CNT increases the surface area and improves the absorption potential through van der Waals interactions with hydrophobic compounds emitted through the bacterial culture medium. Fig. 4d represents the EDX data of DVB/PDMS/CNT-coated patches with 12.83% of carbon, 8.17% of oxygen and 0.82% of silicone, suggesting the coating of the polymer on the paper-based microextraction patches. The presence of silicone in the EDX data was due to the use of PDMS during the fabrication of patches.

**Fig. 5:**
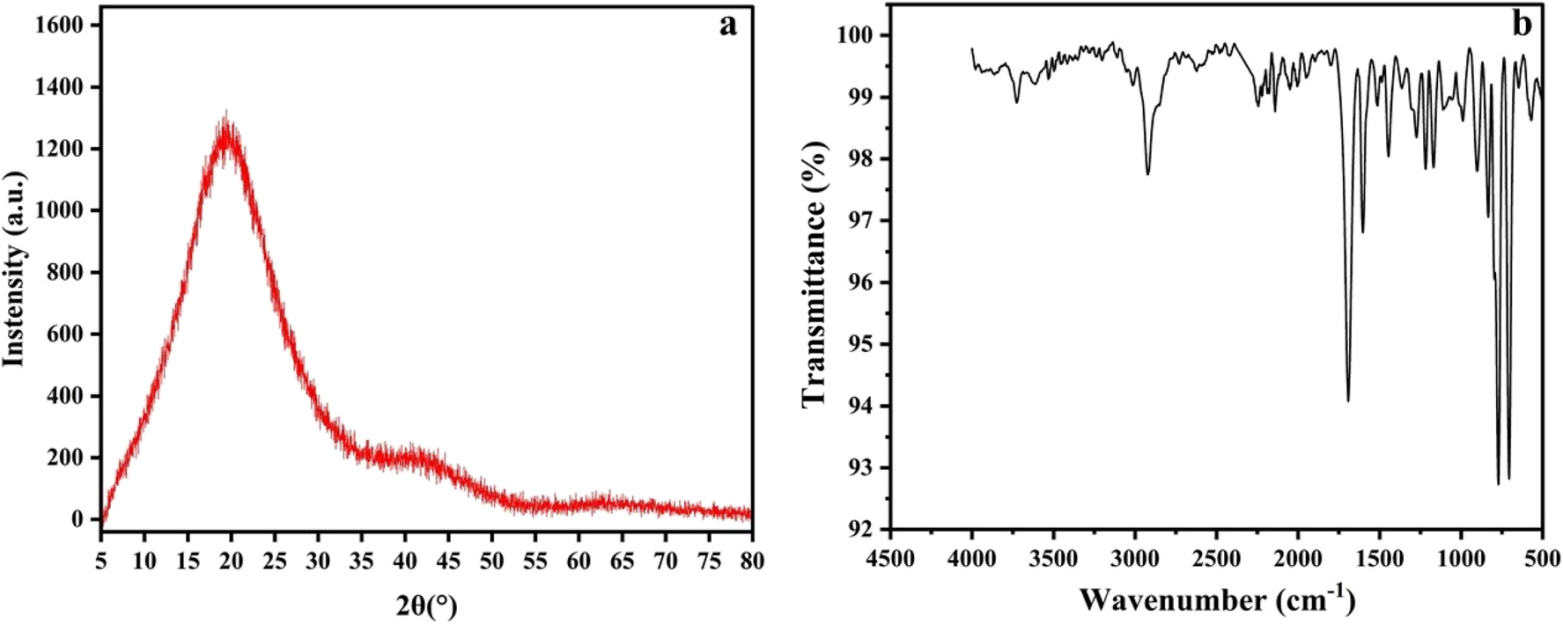
Characterization of our synthesized DVB particles by (a) XRD and (b) FTIR.

The X-ray diffraction (XRD) pattern in Fig. 5a indicates that the material is amorphous and aligned in a cross-linked or polymerized form.^19^ It shows a broad and amorphous peak at approximately 2*θ* ≈ 20°. The FT-IR spectrum in Fig. 5b of the DVB particles for the C–H stretching vibrations (aromatic and vinyl) was observed in the range of 3100–2900 cm^−1^. Additionally, C=C stretching vibrations (aromatic) were detected in the range of 1600–1450 cm^−1^, and C–H bending vibrations (aromatic) at approximately 900–700 cm^−1^, depicting the evidence of the polymer DVB particles.^20^

### 3.1. Stability of p-TF-SPME patches

The thermal and tensile properties of the DVB/PDMS/CNT-coated TF-SPME patches were evaluated using a thermogravimetric analyzer and a universal testing machine.

#### 3.1.1. Thermogravimetric analysis

**Fig. 6:**
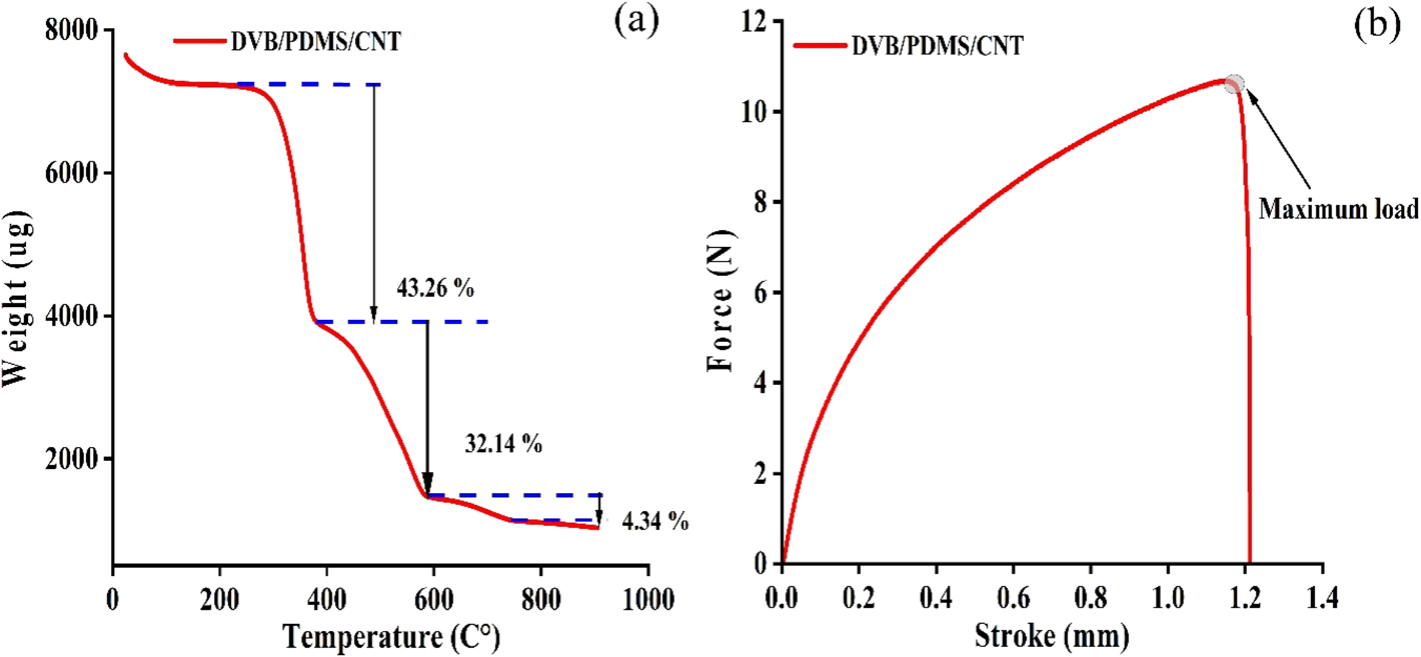
Stability test for the fabricated p-TF-SPME patch through thermal and mechanical stability. (a) Thermal stability data during TGA, and (b) mechanical stability measurement by UTM experiments.

To check the thermal stability, check of the DVB/PDMS/CNT patch, the thermogravimetric analysis (TGA) was performed with 8 mg of patches at a ramp temperature of 5 °C per minute in the temperature range of 30–900 °C. The data demonstrated an initial degradation of the patches started at 230 °C due to the moisture content of the microextraction patch, and it increased up to 376 °C before commencing the second degradation of the tool. This study confirmed that the paper-based patch is stable up to 230 °C for the application as an analytical sample preparation tool. A weight loss of 32.14% occurred between 500 °C and 700 °C, leading to the complete polymer decomposition. The thermally stable carbon nanotube (CNT) material,^21^ which can withstand higher temperatures, accounted for the 4.34% of remaining weight (Fig. 6a).

#### 3.1.2. Universal testing machine analysis

To verify the mechanical stability of the DVB/PDMS/CNT-coated patches, a UTM analysis was performed with a piece of paper patch. The result depicted in Fig. 6b showed the good mechanical stability of our fabricated p-TF-SPME with a mechanical tolerance up to 10.6 N at a 1.2 mm stroke before it could break during the UTM analysis. The presence of CNT improved the tensile property of the patches, whereas the elastic nature of PDMS allowed flexibility.^22^ The DVB material provides cross-linking strength to the p-TF-SPME patches.

### 3.2. Validation of the p-TF-SPME patch and greenness of the proposed technique

**Fig. 7:**
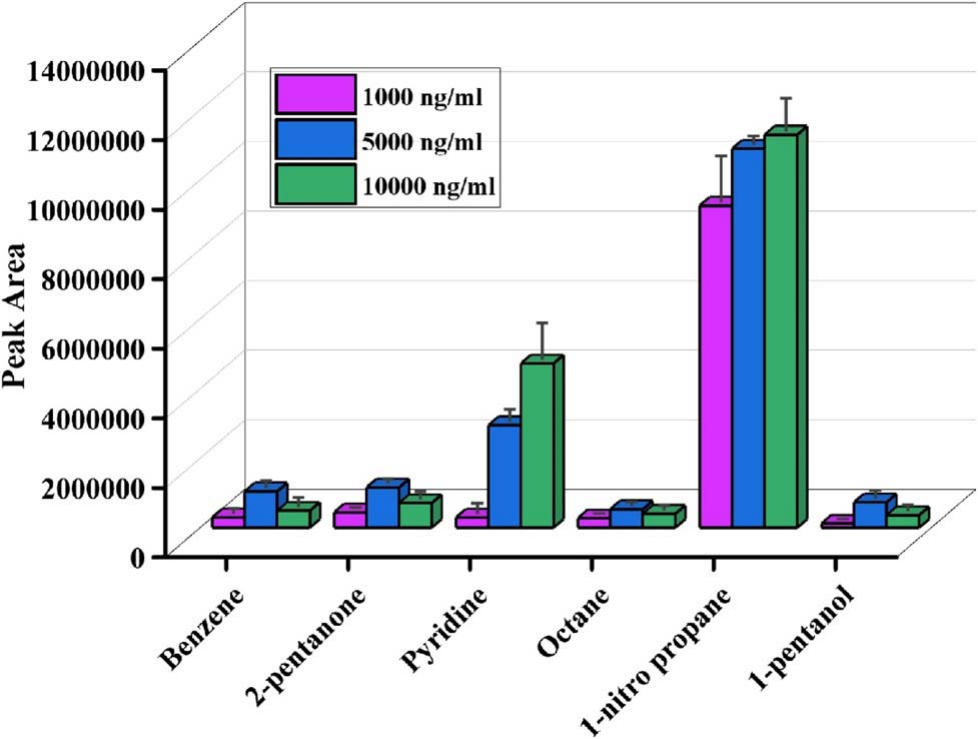
Headspace validation of p-TF-SPME by McReynolds standard solution.

To investigate the extraction efficiency of patches for headspace extraction, the p-TF-SPME patches were exposed to the headspace of the vials containing the McReynolds standard compounds at three concentrations (1000, 5000, and 10000 ng mL^−1^). Fig. 7 demonstrated that the extraction ability of the patches increased with the increment of the concentration of analytes present in the McReynolds standard mixture. Interestingly, the patches were able to extract 1-nitro propane and 1-pentanol compounds than other analytes from McReynolds standard. This phenomenon could be justified by the high volatility of those analytes and, therefore, the preferable high enrichment of the analytes in the headspace over the liquid matrix. This investigation suggested that the fabricated p-TF-SPME patches were able to extract analytes from the headspace matrix of the standard solution.

To validate the p-TF-SPME patch for direct immersion study, we immersed the patches into three individual concentrations of octane in the range of 1000–10 000 ng mL^−1^. Octane was selected for the validation of the patches as it is a moderately volatile and straight-chain hydrocarbon, thus making it suitable as a representative analyte for many VOCs present in biological systems.

**Fig. 8:**
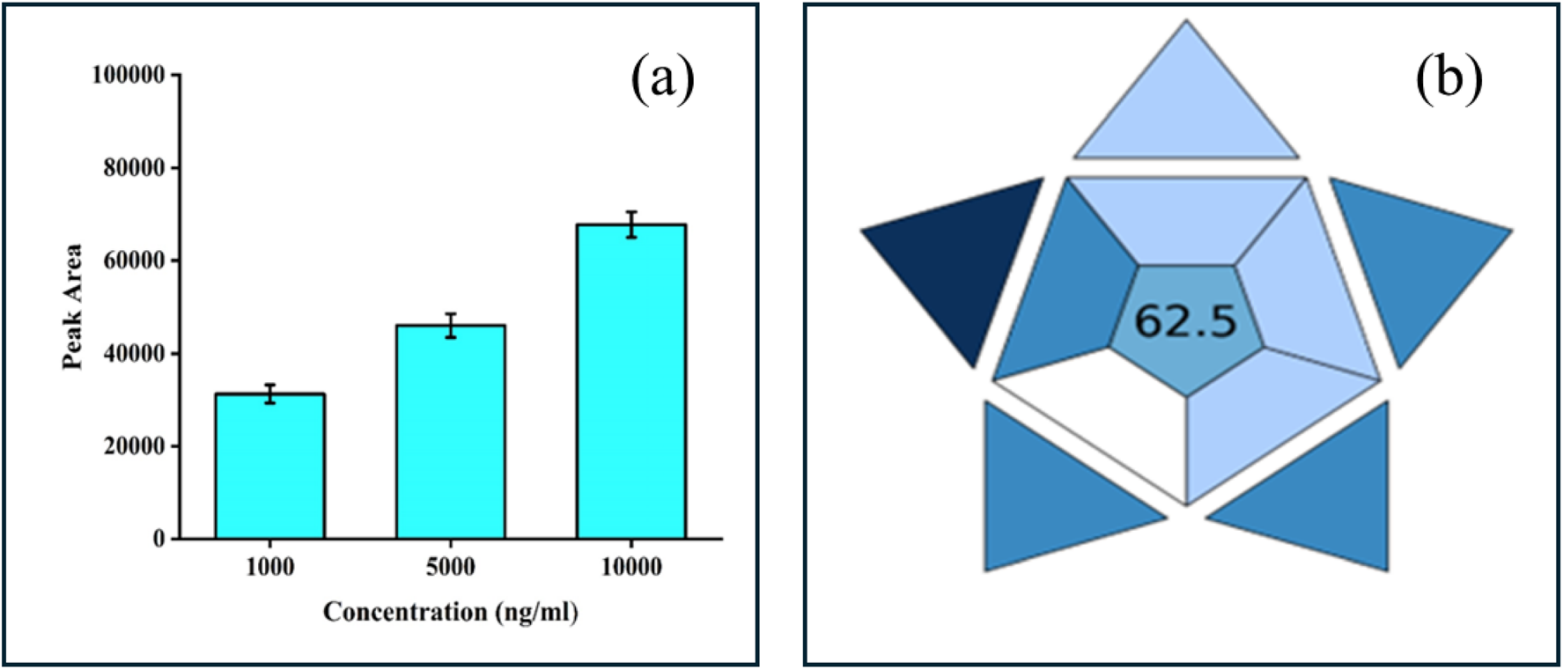
(a) Validation of the fabricated p-TF-SPME patch using the octane standard solution at various concentrations, (b) estimation of BAGI score to determine the greenness of the technique.

The paper-based patches were directly immersed in the solution for 30 minutes to extract the analytes. Later, the patches were desorbed in ethylacetate for 10 minutes for mass transfer from the patches to the solution matrix, and then the analyte was measured by GC-MS. This study demonstrated the increase in extraction efficiency of p-TF-SPME with an increase in the concentration of the standard solution (Fig. 8a). This study suggests that our laboratory-designed p-TF-SPME is suitable for the extraction of VOCs from biological matrices.

To check the greenness of the technique, the BAGI score was calculated based on several parameters (ESI Table 1†) of the sample preparation during the characterization of *Staphylococcus* species. Here, the BAGI score was estimated to be approximately 62.5, indicating the eco-friendliness of this proposed technique employing the p-TF-SPME patch (Fig. 8b). This score indicates that the proposed technique may be considered as a green sample preparation approach for the detection of bacterial pathogens.

### 3.3. Extraction of McReynolds standard by p-TF-SPME and commercial patches

**Fig. 9:**
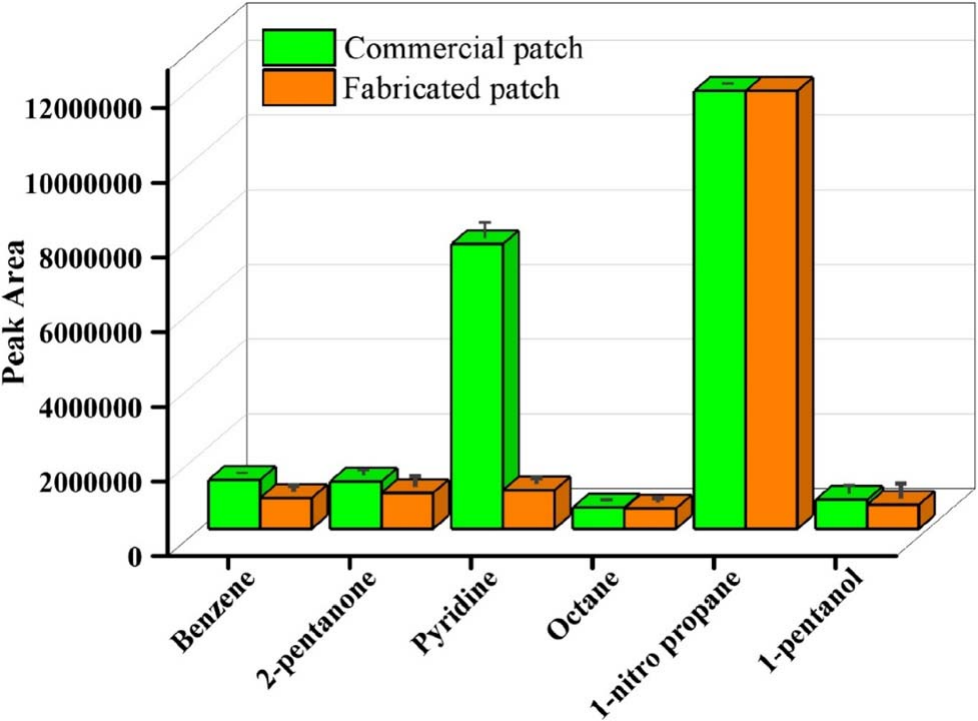
Investigation with p-TF-SPME and commercial patches for extraction of McReynolds standards.

To validate the fabricated paper-based microextraction patches, McReynolds standards were used at the concentration of 1000 ng mL^−1^, and extractions were performed for 1 hour using the p-TF-SPME and commercial DVB/PDMS patches. The Fig. 9 demonstrated that the p-TF-SPME patches were able to extract all the analytes from the McReynolds standards solution. However, the commercial TF-SPME patches exhibited higher extraction efficiency than the paper-based TF-SPME.

### 3.4. Volatile emission from bacterial culture media

**Fig. 10:**
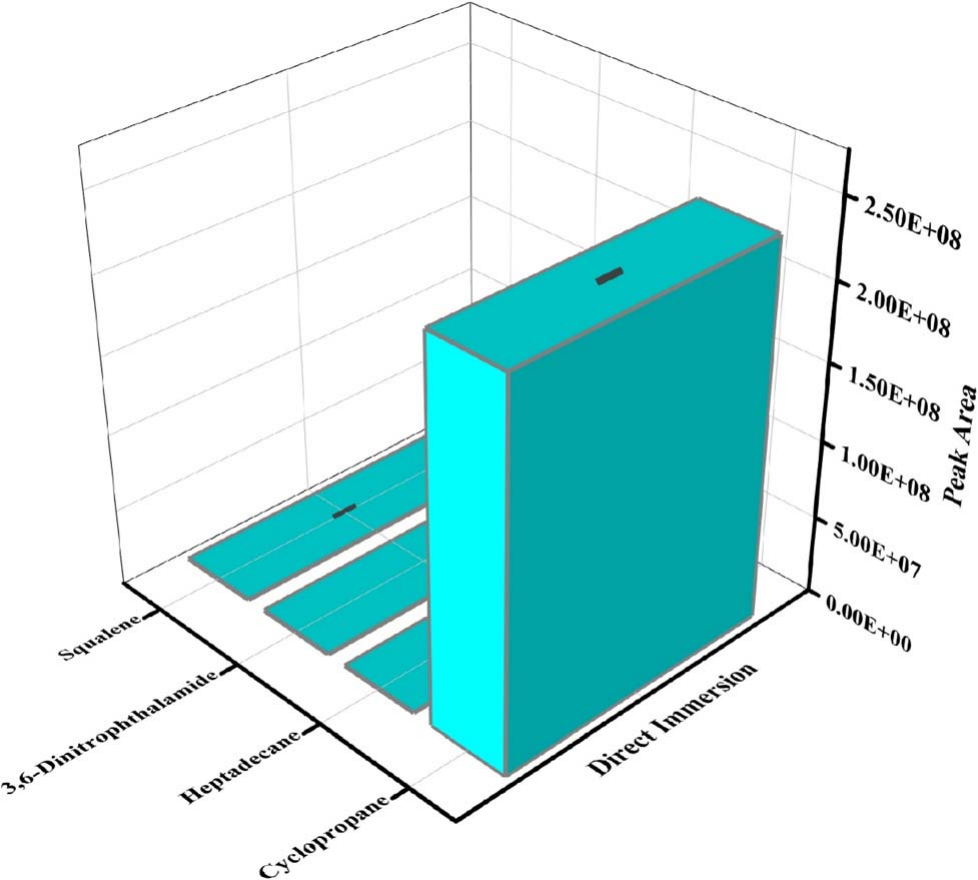
Volatile emission from *Staphylococcus* culture media during direct immersion of p-TF-SPME patches.

**Fig. 11:**
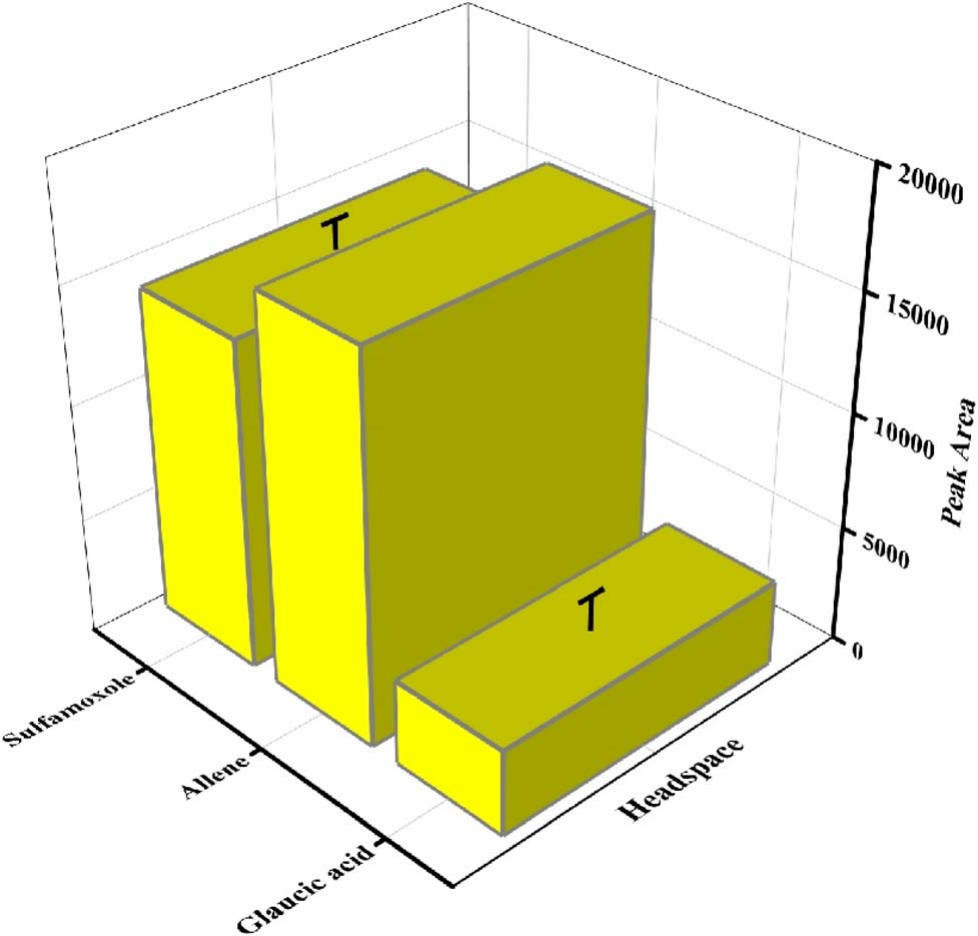
Volatile emission from *Staphylococcus* culture media during headspace extraction by p-TF-SPME patches.

The p-TF-SPME patches were introduced into bacterial culture media to extract of metabolites during the bacterial growth phase. This study demonstrated the presence of seven volatile compounds during *Staphylococcus* bacterial growth phase in culture media as compared to the control matrix (the uninoculated sample media). During the direct immersion of the p-TF-SPME tool, four metabolites including cyclopropane, heptadecane, 3,6-dinitrophthalamide and squalene were observed during the volatile emission from the culture media (Fig. 10). Furthermore, metabolites including sulfamoxole, glaucic acid and allene were obtained from the headspace of the culture vial of *Staphylococcus* bacterial pathogen (Fig. 11). A few common compounds were observed in both headspace and bacterial culture medium.

### 3.5. Chemical interaction and pathophysiology of the volatile emission from culture matrix

The efficiency of DVB/PDMS/CNT-coated TF-SPME patches for the extraction of volatile and semi-volatile compounds from the culture media could be justified through the various interactions among sorbent particles on microextraction patches and the compounds emitted from the culture media. The extraction of cyclopropane by the patches was attributed due to the van der Waals and hydrophobic interactions among CNT-blended PDMS sorbent materials with the analytes emitted from the culture media. Squalene, a non-polar terpene compound with conjucated double bonds, interacts with PDMS through van der Waals forces and exhibits weak π–π stacking with MW-CNT. Furthermore, the presence of moderately polar DVB in the coating recipe of p-TF-SPME, facilitated the preconcentration of glaucic acid through the hydrogen bonding and dipole–dipole interactions. The analytes allene was immobilized on our designed p-TF-SPME patches due to the hydrophobic interaction with PDMS material. The extraction of sulfamoxole by microextraction patches could be justified by the strong interaction of DVB particles with the analyte through the hydrogen bonding and dipole–dipole interactions.^23^ In addition, Henry's law constant plays^24^ an important role in the distribution of pathogen-emitted compounds to the headspace and solution matrix of the culture vial. As per law, a low Henry's partition coefficient suggests a preference for the gas phase enrichment of volatile compounds, indicating the presence of cyclopropane and allene in headspace of the bacterial culture matrix. The glaucic acid with a relatively high Henry's partition coefficient was observed to remain in the liquid phase of the culture medium. In this study, the metabolites associated with the bacterial species were extracted in the growth phase of the bacterial species due to the major enrichment of metabolites during this phase.

Squalene, an important component in the physiology of *Staphylococcus aureus*, is primarily involved in staphyloxanthin biosynthesis in *Staphylococcus aureus,* contributes to the integrity of the cell membrane.^25^ Furthermore, staphyloxanthin is responsible for protecting the bacteria from the host environment. The microbial volatile organic compound database also revealed the presence of allene in pathogen media.^26^ The emission of cyclopropane from microbial culture was reported in the case of *E. coli* bacteria due to the presence of the CFA gene (in *E. coli*), associated with the production of cyclopropane fatty-acyl-phospholipid synthase (CFA).^27^ The presence of heptadecane was also reported as a bacterial metabolic marker compound by previous investigators.^28^

## 4. Conclusion

This study described the fabrication of a disposable paper-based thin film solid-phase microextraction tool for monitoring of volatile emission to identify *Staphylococcus aureus* bacterial species. The efficiency of a simple paper-coated TF-SPME patch for capturing semi-volatile and volatile compounds is well established by this study. The sorbent material was selected based on its compatibility with the paper substrates. Hence, the selectivity of the technique can further be optimized using appropriate polymers and nanocomposites depending on the analytes of interest. The p-TF-SPME offers several advantages, including biodegradation of the material, environmental sustainability, simple fabrication method, minimal solvent usage and convenient sample preparation method. The fabricated patch was able to compete with the commercial patches for in extracting all the compounds used in the validations. In addition, the BAGI score suggests that the technique is eco-friendly for clinical diagnosis. Therefore, it may contribute to the green analytical approach, and suggests a wide application in clinical diagnosis for rapid screening in the mass population, particularly in respiratory infections. However, additional research is necessary to evaluate this method using real clinical samples from patients. The study was conducted on a single bacterial *Staphylococcus* strain. Future studies may be conducted with multiple bacterial strains and clinical isolates to get a broad range of metabolites. Finally, this study may be considered as an alternative technique for the identification of bacterial species to the traditional prolonged culture-based study, where it takes almost 48–72 hours to grow the bacteria in the culture plate. Therefore, this study may facilitate the shortening of the diagnosis time of *Staphylococcus* bacterial species in biological samples, and this may help clinicians to switch from broad-spectrum antibiotics to narrow-spectrum antibiotics during antimicrobial resistance, where the knowledge of bacterial species is important. Furthermore, the fabrication of the p-TF-SPME technique utilizes biodegradable materials, which significantly reduces the chances of environmental pollution. This technique minimizes solvent usage, facilitates resource conservation and reduces the risks associated with solvent disposal. Apart from these, the fabrication cost of the paper-based patch on the laboratory scale is less than the available price of commercial TF-SPME devices. This proposed method, coupled with a portable mass analyzer, could be used for the rapid determination of respiratory infection in remote healthcare settings for future applications. Therefore, our proposed paper-based patches may be fabricated in a cost-effective way. Future research may be considered in designing a pathological green sampling kit to characterize the pneumonia-causing *Staphylococcus* bacterial infection.

## Data availability

The authors confirm that the data supporting the findings of this study are available within ESI.†

## Conflicts of interest

The authors declare no conflict of interest from the study.

## Supplementary Material

RA-015-D4RA09099C-s001
