# Supplementary material for: Fabrication of disposable microextraction analytical tool for in vitro detection of Staphylococcus bacterial pathogen using volatile metabolites emission
Source: RSC Adv. 2025 Apr 10;15(15):11391–400. doi: 10.1039/d4ra09099c (PMC11984458; doi:10.1039/d4ra09099c)
Supplement: RA-015-D4RA09099C-s001 [file RA-015-D4RA09099C-s001.pdf]

Supplementary Information (SI) for RSC Advances.
This journal is © The Royal Society of Chemistry 2025

Supporting Materials

Fabrication of disposal microextraction analytical tool for in-vitro detection of *Staphylococcus* bacterial pathogen using volatile metabolites emission

Keerthana Selvamuthukumar[a], Debsmita Mandal[a], Harshika Poojary[a], Gouri Illanad[b], Bharath Prasad AS[c], Sophia Koo[d,e,f] and Chiranjit Ghosh* [a,d,e]

[a] Department of Biotechnology, Manipal Institute of Technology (MIT), Manipal Academy of Higher Education, Manipal (MAHE), Karnataka, 576104, India.

[b] Department of Biotechnology, KLE Technological University, Hubballi, Karnataka, 580021, India

[c] Department of Public Health Genomics, Manipal School of Life Sciences (MSLS), Manipal Academy of Higher Education, Manipal (MAHE), Karnataka 576104, India.

[d] Division of Infectious Diseases, Brigham and Women's Hospital, 181 Longwood Avenue, MCP642, Boston, MA 02115

[e] Harvard Medical School, Harvard University, 25 Shattuck Street, Boston, 02115, MA, USA

[f] Dana-Farber Cancer Institute, 450 Brookline Avenue, Boston, MA 02215, USA

* **Corresponding Author:** Chiranjit Ghosh

Email: chiranjit.ghosh@manipal.edu

1. Fundamental theory of direct immersion and headspace mode in SPME

In the past few years, significant advancements have been made in solid-phase microextraction and thin-film microextraction, which are considered the popular non-exhaustive and passive sampling techniques. The SPME technology works on the partition equilibrium between the sample matrix and the sorbent coating[1]. The main operational principle to quantitate the extracted compounds by the following procedure can be determined through the equation 1:

$$n_e^{eq} = \frac{K_{es}V_eV_s}{K_{es}V_e + V_s}C_s^0$$ ------- (1)

where n= quantity of analytes extracted, Ve= Volume of the extraction phase, vs= sample volume, Kes = The distribution constant of the analyte in the extraction phase to the sample matrix and Cs = sample concentration.

There are two common extraction modes in the SPME technique: direct immersion (DI-SPME) and headspace analysis (HS-SPME). In DI-SPME, the sorbent-coated fiber is directly immersed in the sample matrix, facilitating mass transfer from the sample matrix to the SPME-coated fiber.

Equation (2) depicts the DI-SPME in the condition when the sample volume (VS) is much higher than the Kes and Ve

$$n = K_{es}V_eC_s$$ ---------- (2)

In case of the headspace analysis, the fiber is exposed to the headspace, which consists of volatile and semi-volatile compounds present above the sample matrix. The equation for the headspace is represented in the equation 3.

$$n = \frac{K_{es}V_eV_s}{K_{es}V_e + V_s + K_{hs}V_{hs}}C_s$$ ---------- (3)

Where Khs represents the distribution constant available between the sample matrix and the headspace, Vhs is the volume of the headspace (negligible).

The mode of extraction used in the study will be chosen based on the researcher's requirements. For instance, DI-SPME enhances the extraction efficiency, particularly for the polar and semi-volatile compounds, making it resourceful for the untargeted analysis for capturing innumerable compounds. The HS-SPME can be used in situations where direct contact between

the sample and the SPME device should be avoided to reduce the fouling and extend the lifespan of the coating[2].

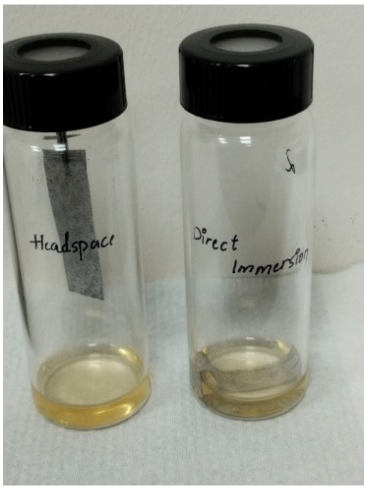

Figure S1: Direct immersion and headspace extraction of metabolites from *Staphylococcus aureus* culture media

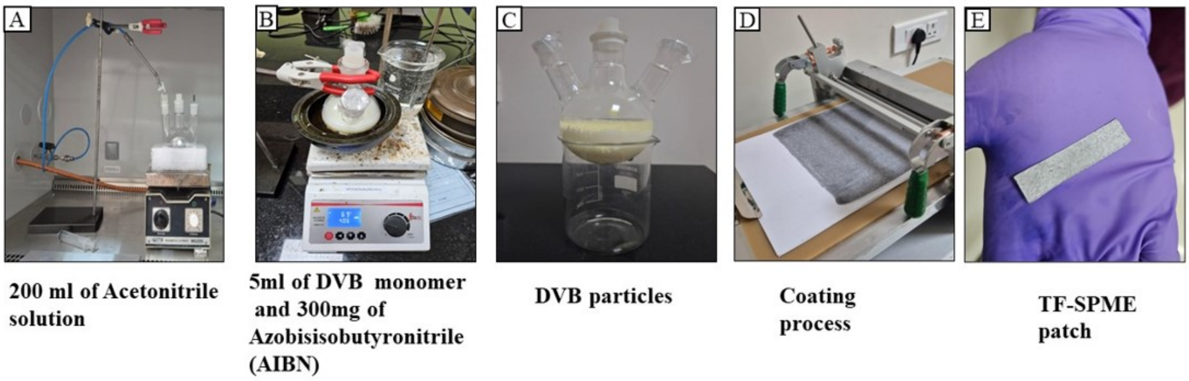

Figure S2: Fabrication of p-TF-SPME patch from the synthesized DVB polymer

Table S1: The parameters associated with the BAGI score[3]

S. No	Parameters	Factors considered	Justification
1	Type of Analysis	Screening	Our study relies on untargeted analysis
2	Multi- or single-element analysis	Multi-element methods that are used for the determination of 6–15 analytes of the same chemical class or 2–15 analytes of different chemical class	We were able to determine 8 compounds of different classes through the direct immersion and headspace mode of extraction and later analysis through the GC-MS
3	Analytical technique	Instrumentations that are not commonly available in the laboratories	We used GC-MS/MS which are not commonly available in the labs.
4	Simultaneous sample preparation	2-12	We were able to perform 10 samples at the same time for the sample preparation.
5	Sample preparation	Miniaturized extraction sample preparation technique	We used a miniaturized extraction technique based on SPME
6	Samples per h	2 hours	1.5 hours for achieving O.D<br>30 minutes for the desorption
7	Reagents and materials	Commonly available reagent Acetonitrile (ACN) was used	We used for 1ml of acetonitrile for the desorption of the metabolites that were

			extracted from the p-TF-SPME patch
8	Preconcentration	One step preconcentration step is required to attain the sensitivity	We incorporated TF-SPME based approach for the preconcentration
9	Degree of automation	Semi-automated method with the common device	It is a semi-automated analysis through GC-MS/MS
10	Amount of Sample	100-500 µL (or mg) bioanalytical samples	Only 300 µL of bioanalytical samples were utilised for the analysis

References

1 L. Cai, J. Dong, Y. Wang and X. Chen, *Electrophoresis*, 2019, **40**, 2041–2049.

2 E. Gionfriddo, É. A. Souza-Silva and J. Pawliszyn, *Anal Chem*, 2015, **87**, 8448–8456.

3 N. Manousi, W. Wojnowski, J. Płotka-Wasylka and V. Samanidou, *Green Chemistry*, 2023, **25**, 7598–7604.
